# Quality of life and psycho-emotional wellbeing in bladder cancer patients and their caregivers: a comparative analysis between urostomy versus ileal orthotopic neobladder

**DOI:** 10.3332/ecancer.2021.1163

**Published:** 2021-01-05

**Authors:** Marianna Masiero, Derna Busacchio, Paolo Guiddi, Paola Arnaboldi, Gennaro Musi, Ottavio De Cobelli, Florence Didier, Gabriella Pravettoni

**Affiliations:** 1 Applied Research Division for Cognitive and Psychological Science, European Institute of Oncology, IRCCS, Milan, Italy; 2 Department of Oncology and Hemato-Oncology, University of Milan, Milan, Italy; 3 Medical Psychiatry and Medical Psychology Service, Cantonal Socio-Psychiatric Organisation (OSC), Lugano, Switzerland; 4 Unit of Neoplasms of the Male Genital Apparatus, European Institute of Oncology, IRCCS, Milan, Italy; 5 Division of Urology, European Institute of Oncology, IRCCS, Milan, Italy

**Keywords:** radical cystectomy, neobladder, urostomy, decision-making, caregivers, personality

## Abstract

**Background:**

The impact of neobladder and urostomy on bladder cancer patient’s health-related quality of life (HR-QoL) is controversial and many issues currently remain under-investigated. Initial studies pointed out that the emotional responses of caregivers might be ‘*contagious*’, influencing emotional reactions in bladder cancer patients undergoing radical cystectomy.

**Methods:**

Three hundred and eighty-two bladder cancer patients (aged *M* = 67.29 years; SD = 9.23) (16.9% (65) were female and 82.9% (319) were male) and their caregivers were enrolled. Data were collected prospectively: at T0 (1 month before the surgery), at T1 (2 weeks after the surgery, at patient discharge from the hospital) and at T2 (6-month follow-up). At each time point (T0, T1 and T2), a set of questionnaires (EORT QLQ-C30 and emotion thermometer) were given to patients and their caregivers.

**Results:**

All patients reported a general improvement in the HR-QoL and global health status/QoL from T0 to T2 (*p* < 0.001). No significant differences were observed between neobladder and urostomy. At T0, the emotional thermometer total scoring in caregivers was positive in relation to HR-QoL (*p* < 0.001) and negative in relation to the patient’s perception of QoL (*p* < 0.001) and global health (p < 0.001). Similar trends were observed at T1 and T2.

**Conclusions:**

These results suggest that patients and their caregiver’s emotional reactions to cancer are deep-rooted and strongly interconnected, and they provide innovative insights for the clinical management of bladder cancer patients.

## Background

Conserving quality of life (QoL) and handling symptoms of cancer treatment are essential aspects in cancer clinical care [1, 2]. This is particularly true for life-threatening diagnoses [3–7], such as bladder cancer, where patients who undergo radical cystectomy experience several physical symptoms, as well as significant social, cognitive, functional and relational dysfunctions and emotional distress [8, 9]. Currently, the standard surgery therapy for treating patients with bladder cancer is radical cystectomy with urinary diversion: urostomy versus ileal orthotopic neobladder [10–13]. Radical cystectomy is a surgical option when other methods, i.e., chemotherapy, radiotherapy and transurethral resection of bladder cancer, fail. Patients usually undergo radical cystectomy at least a month after cancer diagnosis and they are often psychologically and physically exhausted with pain and discomfort. Growing evidence [11, 13–17] reported that neobladder and urostomy surgeries have different physical, functional and psychological consequences. Consistently, patients with neobladder reported problematic and urinary incontinence [14, 15], requiring a significant change in lifestyle (e.g., setting alarms to go off during the night and day in order to guarantee regular voiding, wearing pads and changing underwear) [8] and habits [16, 17]. Otherwise, patients with urostomy face complicated conduit options, such as stoma, catheters and related visual stigma [18, 19]. Furthermore, women reported problems associated with orgasms (which may be caused by a reduction in vaginal endoluminal volume and damaging of the vascular flow), while men reported erectile dysfunction (which may be caused by the damaging of the sympathetic and parasympathetic nerves), and both experienced an important reduction in their libido affecting their relationship with their partner [20]. Several studies highlighted that while physical and functional tasks recovered after 1 year from the end of treatment [1], the psycho-emotional burden associated with the consequences of the surgery remained [12, 21, 22]. However, the long-term impact of the neobladder and urostomy on patients’ health-related QoL (HR-QoL) is controversial and many issues currently remain under-investigated [13, 21]. For example, a meta-analysis on QoL after radical cystectomy [23] reported no difference between neobladder and urostomy on patients’ mental and social health. Furthermore, other authors reported a significant improvement in physical health in both types of surgery. Consistently, some studies [18, 22], for example Hedgepeth *et al* [18], reported no differences in functional outcomes, such as body image and urinary, sexual and bowel functions. Other studies, such as Philip *et al* [10], observed that the neobladder allowed patients to improve their QoL, helping them to recover from the surgery more quickly, leading to a relatively normal lifestyle.

Overall, it seems to emerge from the literature that similar to what happens with other major surgical procedures in cancer care, such as pelvic exenteration, patients show a long-term positive adjustment even when faced with radical changes in daily routine and body image perception [24]. Existing studies focused exclusively on patient perspectives [13, 18, 19, 21, 25], without considering other possible moderator factors, such as emotions experienced by people who are close to the patients. Specifically, to the authors’ knowledge, no studies are available about how the personal experience of the disease of the caregivers might affect patient’s adjustment and recovery after radical cystectomy surgery (neobladder and urostomy). Normally, the caregiver is actively involved in home medical treatments, delivering almost 60% of the medical and nursing care (e.g., providing medications, scheduling clinical appointments, follow-ups and routine medical examinations), spending approximately 32.9 hours per weeks in caring activities, and becoming an essential emotional and functional support for the patient [25]. A growing body of studies pointed out that the caregiver and patient’s psycho-emotional wellbeing are strictly interrelated, and might foster patient’s long-term adjustment [26] and resilience to the cancer [27]. Coherently, Hodges *et al* [28] reported a positive association between the patient’s and caregiver’s distress and observed that ‘*emotional wellbeing of one family member affecting the others*’. The close association between patient’s and caregiver’s distress suggests ‘*that is if one partner were to become distressed, it is more than likely that the other partner will do so also*’ [28 page 9]. More in general, considering the entire family system of the patient, Faccio *et al* [29] highlighted the pivotal role of the family-centred models of care as significant others’ coping strategies that may booster adjustment to the disease and well-being of the cancer patients through the disease trajectory. Consistently, Litzelman *et al* [30] found that patients with caregivers who have higher depression symptoms perceived to receive a poor quality of care by healthcare professionals. On the contrary, a caregiver’s wellbeing is strictly correlated with the higher perception of the quality of the care in patients. Furthermore, cancer symptoms’ perception in patients is positively related to the emotional wellbeing and physical function in their caregivers [30]. Some authors explained this association suggesting that caregivers who feel emotional distress and/or poor physical health may have a diminished capacity to support patients in the care process, i.e., attending clinical visits, providing medical treatment and supporting the patient in understanding medical instructions and in care decision-making [30, 31].

In this vein, we argue that the emotional state of caregivers might be “*contagious*’, influencing the emotional reactions in patients. This approach highlighted that patients and their caregivers experience cancer as a dyad impacting each other [32–34]; coherent to this, Sterba *et al* [31], in a study on head–neck cancer patients and their caregivers, affirmed that patient’s adjustment might be influenced by how patients and their caregivers move through the disease pathway.

Nevertheless, caregivers’ burden in bladder cancer patients is not well investigated and studies on caregiver’s perception of patient well-being are scarce and limited [1, 35].

With this in mind, the main aim of this study was twofold. Firstly, to assess the differences in the QoL and the psycho-emotional wellbeing of patients with neobladder and urostomy. We hypothesised that, immediately after the intervention, patients with the urostomy feel more discomfort and emotionally burdened than patients with a neobladder; but this difference will reduce with time (after 6 months of surgery). Secondly, to analyse the physical and psycho-emotional wellbeing in patients and their caregivers through the disease trajectory (from the initial diagnosis to the follow-up phase). We hypothesised that better patient adjustment to the consequences of the radical cystectomy (neobladder and urostomy) might be associated with the psycho-emotional wellbeing in the caregiver.

## Method

### Participants and procedure

The current observational study was carried out within the Urology Division and Psycho-oncology Division of the European Institute of Oncology (Italy) from 2016 to 2020 (the study is ongoing). A convenience sample of 382 bladder cancer patients (aged *M* = 67.29 years; SD = 9.23, ranging from min. 38 to max. 90) (16.9% (65) were female and 82.9% (319) were male) and their caregivers were enrolled. The primary caregivers were mainly members of the close family (respectively: 63.8% was wife/husband, 19% was daughter/son, 1.8% was sister/brother, 2.9% were other family members and 0.3 were friends). All participants who enrolled had an initial bladder cancer diagnosis confirmed histologically and were eligible for radical cystectomy with ileal conduit construction (neobladder) or urinary diversion (urostomy).

*Inclusion criteria* were (a) for bladder cancer diagnosis; (b) no severe cognitive or psychiatric impairment and (c) written informed consent. Data were collected prospectively: at T0 (1 month before the surgery), at T1 (2 weeks after the surgery, at patient discharge from the hospital) and at T2 (6-month follow-up). At each time point (T0, T1 and T2), a set of questionnaires were given to patients (EORT QLQ-C30 and emotion thermometer) and their caregivers (emotion thermometer).

Patients and their caregivers were enrolled by an endoscopy nurse during the standard consultation for the surgery, in which patients and their caregivers received all clinical information about radical cystectomy (clinical features, procedures and consequences). All eligible participants received a detailed presentation of the study. Those wishing to participate read and signed a written consent form. Participants were volunteers, and they could withdraw their consent at any point during the study.

At all time points, patients received psychological counselling in order to measure and monitor psychological wellbeing, QoL and HR-QoL. At T0 and T1, the psychological counselling was carried out within the hospital, while at T2 it was carried out by telephone. The study was conducted in accordance with the Helsinki’s Declaration (59th WMA General Assembly, Seoul, 2008).

### Instruments

Emotion thermometer (ET) [31] is a self-administered test used to assess emotional distress in oncological patients. The ET is composed of five visual analogue scales ranging from 0 (none) to 10 (extreme), respectively: stress, anxiety, depression, anger and need for help. Each subscale provides information about emotional burden. The general cut-offs for each visual scale were 0–3 low distress and 4–10 high distress [36, 37]. These values have been recommended by the National Comprehensive Cancer Network (NCCN) [31]. Furthermore, a general value of the emotional burden is obtained by the sum of total scores obtained in each subscale. It is defined as ‘emotional thermometer total scoring’.

Patients are given the question ‘How distressed have you been during the past week on a scale of 0–10?’ The cut-off for this test is usually 3v4 as recommended by NCCN in 2007 [26].

European Organisation for Research and Treatment of Cancer Quality of Life Questionnaire-Core 30 (EORTC QLQ-C30) [38] is a self-administered questionnaire composed of 28 items on a 4-point Likert-type scale (ranging from 1 = ‘Not at all’ to 4 = ‘Very much’); and two items, general global health status and QoL, on a 7-point Likert-type scale (ranging from 1= ‘Very poor’ to 7= ‘Excellent’). Overall, EORTC QLQ-C30 provides information about three different domains: *functional* (respectively: physical, emotional social, role, cognitive and financial) in which high values suggest a healthy level of functioning; *symptoms* (respectively: appetite loss, fatigue, pain, nausea, constipation–diarrheal, dyspnoea and insomnia) in which high values suggest a high level of symptomatology and global health status/QoL, in which high values suggest a high QoL. The values should be transformed from 0 to 100 in order to permit and to compare the QoL between different studies and for a more accessible interpretation of the results. However, rough data may also be used [45, 46].

### Data analysis

Descriptive statistics are used to depict the characteristics of the sample. Consistent with our primary and secondary aims, a series of repeated measures (analysis of variance with mixed-designs) (within-subjects variables and between subjects variables) were implemented: firstly, to assess general global health status and QoL (measuring by EORTC QLQ-C30) and psycho-emotional wellbeing (measuring by ET) (*dependent variables*) at all time points (*within-subject factors* ) (at T0: 1 month before the surgery; at T1: 2 weeks after the surgery, at patient discharge from the hospital and at T2: 6-month follow-up) between patients with neobladder or urostomy (between-subjects factor); secondly, to assess the differences in psycho-emotional wellbeing (ET) of the caregivers (*dependent variables)* at all time points (*within-subject factors* ) between patients with neobladder or urostomy (*between-subjects factor*). For the EORTC QLQ-C30, two types of analysis were conducted. Consistent with the EORTC QlQ-C30 guidelines [39], a linear transformation of the data (0–100) was conducted in order to increase the possibility to compare our results with the results observed in other studies. Secondly, we decided to use also the rough data, without transformation as reported by other studies [40].

Considering the high correlation between items, we created a new variable named ‘*HR-QoL*’ adding items from 1 to 28 (functional and symptoms scales), in which high values meant a worst health-related QoL. On the other hand, items 29 and 30 were used singularly to define, respectively, *global health status* and *QoL*, in which high values meant a better health status and QoL. This permits us to consider separately individual evaluations of global health status and QoL.

Finally, for each assessment point (T0, T1 and T2) the analysis was performed considering only subjects with all time points completed (listwise deletion strategy). Furthermore, when Mauchly’s test of sphericity indicated that the assumption of sphericity was violated, Greenhouse and Geisser and Huynth–Feldt corrections were used according to the *ε* value [41].

Finally, a correlational analysis using Pearson’s correlation coefficient was conducted to assess the association between psycho-emotional wellbeing in patients and caregivers. Data were analysed using SPSS (IBM, USA) version 26.0.

## Results

### EORTC QLQ-C30

#### Global health status, QoL and HR-QoL

Consistent with the global health status (GHS), patients reported a reduction in the GHS from T0 to T1 and an improvement from T1 to T2 (*F*(2, 198) = 15.734, *η*^2^ = 0.126, *p* < 0.001); the same trend was observed for QoL; patients reported a reduction in the QoL from T0 to T1 and an improvement from T1 to T2 (*F*(2, 198) = 10.272, *p* < 0.001, *η*^2^ = 0.094). Furthermore, a new variable named HR-QoL was calculated, in which a lower score suggests a better HR-QoL. Patients reported a significant improvement in the HR-QoL from T0 to T1 and from T1 to T2 (*F*(1.946, 525.374) = 137.181, *p* < 0.001, *η*^2^ = 0. 337) (see Table 1). No significant difference was observed between patients having neobladder or urostomy.

### EORTC QLQ30 – Linear transformation

#### Global Health Status/QoL

For the ‘*GHS/QoL*’, patients reported an improvement from T1 to T2 and from T0 to T2 (*F*(2) = 14.867, *p* < 0.001, *η*^2^ = 0.131). No significant difference was observed between patients having neobladder or urostomy (see Table 2).

### Functional scales

**Figure 1.**
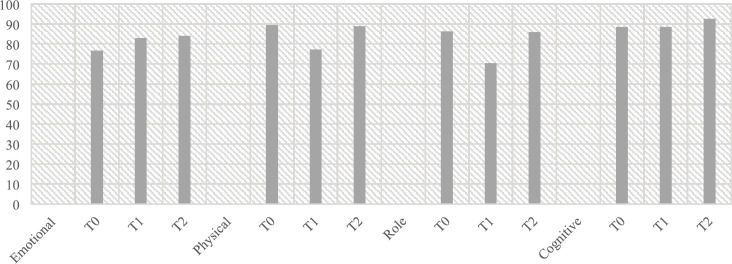
Mean values for functional scales. *T0 Pre-surgery: 1 month before the surgery; *T1 Post-surgery: 2 weeks after the surgery; *T2 Follow-up: 6 months post-surgery.

Patients reported a significant difference in the *emotional scale* (*F*(2, 190) = 9.638, *p* < 0.001, *η*^2^ = 0.092), observing an improvement from T0 to T1 (*p* < 0.003) and from T0 to T2 (*p* < 0.001); the *physical scale* (*F*(2, 188) = 24.770, *η*^2^ = 0.209, *p* < 0.001), observing a reduction from T0 to T1 (*p* < 0.001) and an improvement from T1 to T2 (*p* < 0.00); the *role scale* (*F*(1, 887, 183.925) = 15.311, *η*^2^ = 0.135, *p* < 0.001), observing a reduction from T0 to T1 (*p* < 0.001) and an improvement from T1 to T2; and the* cognitive scale* (*F*(2, 196) = 4.733, *p* < 0.001, *η*^2^ = 0.046), observing an improvement from T0 to T2 (*p* < 0.002) and from T1 to T2 (*p* < 0.022). No significant difference was observed between patients having neobladder or urostomy (Figure 1).

### Symptom scales

**Figure 2.**
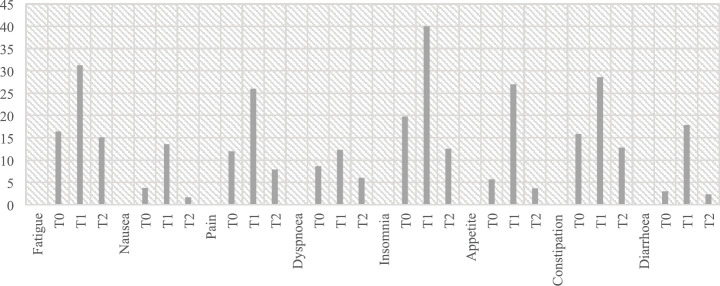
Mean values for symptoms scales. *T0 Pre-surgery: 1 month before the surgery; *T1 Post-surgery: 2 weeks after the surgery; *T2 Follow-up: 6 months post-surgery.

Patients reported a significant difference in *fatigue* (*F*(2, 192)= 29.063, *p* < 0.001, *η*^2^ = 0.232), from T0 to T1 (*p* < 0.001) and from T1 to T2 (*p* < 0.001); *nausea* (*F*(1.480, 146.549) = 19.339, *p* < 0.001, *η*^2^ = 0.163), from T0 to T1 (*p* < 0.001) and from T1 to T2 (*p* < 0.001); *pain* (F(2, 196)= 28.400, *p* < 0.001, *η*^2^ = 0.225), from T0 to T1 (*p* < 0.001) and from T1 to T2 (*p* < 0.001); *insomnia* (*F*(1.838, 181.972) = 32.899, *p* < 0.001, *η*^2^ = 0.249), from T0 to T1 (*p* < 0.001), from T0 to T2 (*p* < 0.028) and from T1 to T2 (*p* < 0.001); *constipation* (*F*(2, 194) = 12.600, *p* < 0.001, *η*^2^ = 0.115), from T0 to T1 (*p* < 0.001) and from T1 to T2 (*p* < 0.001); *diarrhoea* (*F*(1.344, 31.758) = 29.300, *η*^2^ = 0.230, *p* < 0.001), from T0 to T1 (*p* < 0.001), from T0 to T2 (*p* < 0.001) and from T1 to T2 (*p* < 0.001) and *appetite* (*F*(1.475, 144.598) = 37.411, *p* < 0.001), from T0 to T1 (*p* < 0.001) and from T1 to T2 (*p* < 0.001). No significant difference was observed between patients having neobladder or urostomy (Figure 2).

### Patient psycho-emotional wellbeing (ET)

No significant differences were observed between patients with neobladder and urostomy, and thermometer total scoring. Generally, patients reported significant differences at all time points (T0, T1 and T2) in the emotional thermometer total scoring (*F*(1, 596) = 150.307, *p* < 0.001, *η*^2^ = 0.335). In particular, patients reported a significant decrease in the total emotional distress from T0 (*M* = 17.53, SD = 11.25) to T1 (*M* = 3.93, SD = 7.96).

**Figure 3.**
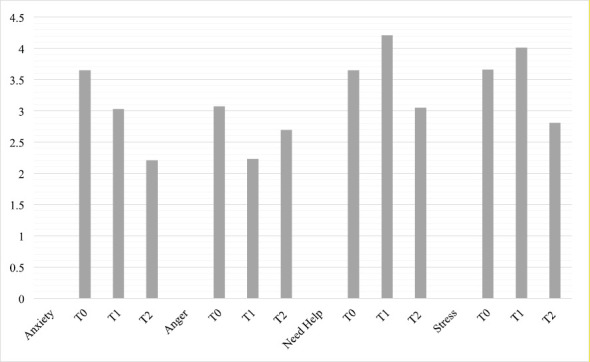
Mean values for need help, stress, anxiety and anger in patients.

Regarding the single visual analogue scales of ET, significant differences were found for anxiety (*F*(2, 198) = 10.754, *p* < 0.001, *η*^2^ = 0.098), stress (*F*(2, 198) = 7.615, *p* < 0.001, *η*^2^ = 0.071), need help (*F*(2, 198) = 6.604, *p* < 0.002, *η*^2^ = 0.063) and anger (*F*(2,198) = 5.298, *p* < 0.006, *η*^2^ = 0.051). In more detail, the level of stress progressively reduced from T0 to T2 (*p* < 0.049) and from T1 to T2 (*p* < 0.001), as did the level of anxiety from T0 to T2 (*p* < 0.001) and from T1 to T2 (*p* < 0.006). For need help, a significant difference was found from T0 to T2 (*p* < 0.001). Anger also decreased from T0 to T2 (*p* < 0.002) (Graph 1). No significant differences were observed between patients with neobladder and urostomy, and the single visual analogue scales of the ET (Figure 3.).

### Caregiver psycho-emotional wellbeing (ET)

Caregivers reported significant differences at all time points for emotional total scoring (*F*(2, 492) = 152.341, *p* < 0.001, *η*^2^ = 0.382). Similar to patients, caregivers reported a higher level of distress immediately after the diagnosis (T0: *M* = 20.46; SD = 11.62) and recovered after 6 months (T2: *M* = 3.72; SD = 9.026). No interaction effect was observed with the type of surgery (neobladder versus urostomy) on the total ET score.

**Figure 4.**
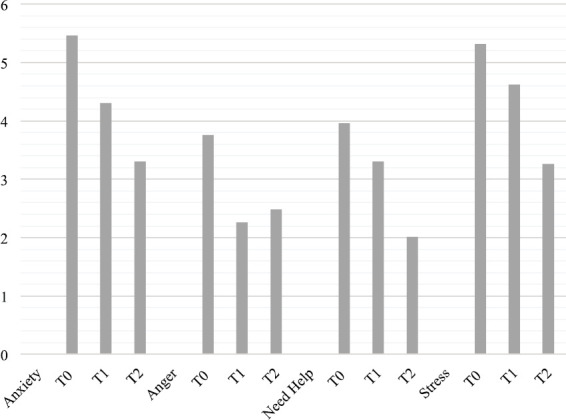
Means for anxiety, anger, need help and stress in caregivers.

Considering the single visual analogue scales of the ET, significant differences were found for stress (*F*(2, 126) = 14.116, *p* < 0.001, *η*^2^ = 0.183), anxiety (*F*(2, 126) = 15.637, *p* < 0.001, *η*^2^ = 0.199), anger (*F*(2, 126) = 6.419, *p* < 0.002, *η*^2^ = 0.092) and need help (*F*(2, 126) = 13.281, *p* < 0.001, *η*^2^ = 0.174). In more detail, stress was very high at T0 and T1, and decreased from T0 to T2 (*p* < 0.001) and from T1 to T2 (*p* < 0.003); need help decreased from T0 to T2 (*p* < 0.001) and from T1 to T2 (*p* < 0.011); anger decreased from T0 to T1 (*p* < 0.011) and from T0 to T2 (*p* < 0.019). Finally, anxiety decreased from T0 to T1 (*p* < 0.005) and from T1 to T2 (*p* < 0.001). No differences were observed between the type of surgery (neobladder versus urostomy) and the single visual analogue scales of the ET (Figure 4).

### Correlation between caregivers psycho-emotional wellbeing and HR-QoL, global health and QoL in patients

#### T0

The emotional thermometer total scoring in caregivers was positively associated with HR-QoL (*r* = 0.359**, *p* < 0.001). This means that a high value of emotional distress in caregivers was associated with a worst HR-QoL in patients. Furthermore, the emotional thermometer total scoring in caregivers was negatively associated to the patient’s perception of QoL (*r* = −0.625**, *p* < 0.001) and patient’s perception of global health (*r* = −0.585**, *p* < 0.001). Thus, a high value of emotional distress in caregivers was associated with a low patient perception of QoL and global health.

#### T1

At T1, the emotional thermometer total scoring in caregivers was positively associated with HR-QoL (*r* = 0.502**, *p* < 0.001) and negatively associated with patient’s perception of QoL (*r* = −0.416**, *p* < 0.001), and patient’s perception of global health (*r* = −0.463**, *p* < 0.001). Similarly, at T2, the emotional thermometer total scoring in caregivers was positively associated with HR-QoL (*r* = 0.606**, *p* < 0.001) and negatively associated with patient’s perception of QoL (*r* = −0.604**, *p* < 0.001) and patient’s perception of global health (*r* = −0.648**, *p* < 0.001) (Table 1).

## Discussion

Overall, this prospective and observational study contributed firstly to better understanding adjustment, psycho-emotional wellbeing and HR-QoL in the context of bladder cancer treatments; and secondly to provide knowledge about the impact of the psycho-emotional well-being on patients undergoing radical cystectomy. In particular, these findings stressed two important points in the care of patients with bladder cancer: firstly, the results reported no significant differences between bladder cancer patients with distinct diversion options (ileal orthotopic neobladder and urostomy) for global health and QoL. This might suggest an adjustment during the disease pathway also for demolitive surgeries, such as urostomy. Secondly, the results suggest an association between psycho-emotional wellbeing in caregivers and physical and psychological outcomes in patients undergoing radical cystectomy with distinct diversion options.

Overall, all patients enrolled in this study reported, after 2 weeks of radical cystectomy, independent type of urinary diversion, a significant decrease in global health status and QoL compared to the pre-hospitalisation assessment. Notwithstanding, after 6 months, all patients (both neobladder and urostomy) recovered progressively, achieving similar values to the pre-hospitalisation, leading to a normal QoL. We presume that this may indicate that initially patients have difficulty in accepting the stoma, but with time they accept it and support adjustment. Immediately after the surgery, patients have little practice in using the stoma and catheters, and need help from their caregivers. Indeed, patients reported a higher level of ‘need for help’ at T1 (2 weeks post-surgery) compared at T0 (6 months post-surgery). Probably, as suggested by Cerruto *et al* [19], over time patients acquired knowledge and expertise in using the stoma, permitting them more independence. Similarly, patients with a neobladder have to progressively adapt to the new situation, which requires a change in their lifestyle due to problematic voiding incontinence [8, 14, 42]. This result is coherent with other studies [24], which reported that no matter how invasive and disrupting the procedure is, patients seem to adjust over time reaching a satisfactory QoL. In particular, Arnaboldi *et al* [24] observed that pelvic exenteration patients, notwithstanding their severe clinical condition and complex surgery, reported adaptive coping strategies and a positive adjustment to their condition.

Similarly, in caregivers, the need to help is higher, at the time of discharge from the hospital. Perhaps, because during the hospitalisation patients and caregivers received brief training from healthcare professionals, whereas at home they have to face the difficulties related to stoma management, with relatively little support from healthcare professionals. With regard to distress, patients and caregivers feel a higher level of emotional distress prior to hospital admission (approximately 1 month before the surgery) and at discharge from the hospital (2 weeks after surgery), but it is reduced after 6 months. Arnaboldi *et al* [43] in a study on bladder cancer patients suggested that patients and their primary caregivers may feel distressed at pre-admission to the hospital because they need more information about their clinical pathway.

Consistently with growing research on cancer caregiving, our data suggest that patient and caregiver’s emotional reactions to cancer seem to be deep-rooted and interconnected [30, 31]. In particular, our data indicates that HR-QoL, Global Health Status, and QoL in patients are associated to psycho-emotional wellbeing in their caregivers at each time point of the disease trajectory (from the diagnosis to follow-ups). Furthermore, this implies that better psycho-emotional wellbeing in primary caregivers might boost not only QoL in patients, but also a series of health outcomes (such as reducing functional impairments and improving symptoms self-management).

Similarly, comparing baseline (T0) with the 6-month follow-up (T2), we found a positive association between stress and need help in caregivers. This might suggest that patients who have a caregiver with a good psycho-emotional wellbeing have an increased probability of better adjustment to the diseases and their physical consequences. We reason also that this might explain the similar trend in adjustment and recovery processes observed in both types of urinary diversion (neobladder and urostomy).

In conclusion, results reported in this study contribute to clarifying the *open debate* regarding the impact of radical cystectomy, neobladder and urostomy, on QoL and psycho-emotional wellbeing of patients by providing some innovative insights for the clinical management of bladder cancer patients undergoing radical cystectomy. This is in line with other studies that have reported a strong association between patient and caregiver emotional reactions [28, 43].

Firstly, we suggest that the experience of demolitive surgeries, such as urostomy, might be integrated and elaborated by the patients if they received adequate emotional support. Secondly, a patient and caregiver supportive relationship might foster physical, social and functional outcomes in the long period, favouring progressive adjustment and recovery. Previous studies [39] have stressed the impact of the physical and psychological status of the caregivers on how cancer patients perceived the quality of the care received by health professionals (e.g., communication with clinicians, care coordination, etc.). Our study additionally takes into account the impact of the psychological wellbeing of the primary caregivers on HR-QoL , global health status and finally QoL of cancer patients, considering both physical and psychological dimensions of the disease.

Thirdly, the association observed between psychological wellbeing in patients and caregivers stressed the need to move beyond viewing patients and caregivers separately, considering psycho-emotional wellbeing concurrently [42].

We think that future psychological interventions for patients undergoing radical cystectomy, with high levels of emotional distress, should be directed and implemented not only to the patient but also to patient–caregiver dyad. In this way, a primary psychological prevention might be provided to reduce the severe emotional burden in bladder cancer patients, improving also physical long-term outcomes.

## Limitations

The study reported some limitations that limited the generazability of the results. One of the major limitations of this study, which may lessen the generalisation of the results, concerns the observational design that does not allow causal explanations of the phenomena observed and poses concerns, in particular, about internal validity. The main limitation of observational studies is the impossibility to randomise participants into different groups. Secondly, during the study, we observed a moderate to high dropout of patients enrolled in the study generating missing data, in particular, from T0 to T2, which probably affected the results and some low-medium effect size reported. The moderate to high dropouts for surgery thereafter decided to continue their post-operative treatments in other clinical centres close to home. Furthermore, the level of distress in our sample (patients and caregivers) is relatively modest. The higher values of emotional burden were observed only at T0 (in both patients and caregivers), immediately after the cancer diagnosis. Future studies will have to overcome the limitations observed. Furthermore, 82.9% of the patients in the sample were male, and their caregivers were their spouses/partners. This factor might have affected the results, considering the ‘gender differences’ in both patient’s cancer adjustment and the caregiving activity. Indeed, female partners perceive more psychological distress and a lower QoL than male partners [44].

## Conclusion

Notwithstanding, to our knowledge, this is the first study conducted using a *joint patient and primary caregiver perspective* in order to understand physical and psychological outcomes (in short term and long term) in bladder cancer patients undergoing radical cystectomy. It provides insights for healthcare professionals to better understand emotional and physical adaptation of patients to the disease and in this case to radical cystectomy and how *disease experience* in bladder patients may be affected by the psycho-emotional wellbeing of their caregivers.

## Funding

No funding.

## Conflicts of interest

The authors declare no conflicts of interest.

## Authors’ contributions

A.P., D.F. and G.P. conceived and designed the experiments. A.P., D.F., D.B., P.G. and M.M. carried out the experiments. M.M. and P.G. analysed the data. D.F, D.B., P.G., M.M., G.M., O. D. and G.P. wrote the manuscript.

## Figures and Tables

**Table 1.** Mean and standard deviation for GHS at each time point (higher score means better global health status), QoL (higher score means better quality of life) and HR-QoL at each time point (lower scores mean better health-related quality of life).

Global health status (GHS)	*M*	SD
GHS – T0	5.24	1.32
GHS – T1	4.84	1.24
GHS – T2	5.63	1.16
***QoL***	***M***	**SD**
QoL – T0	5.34	1.40
QoL – T1	4.70	1.43
QoL – T2	5.44	1.27
**HR-QoL**	***M***	***SD***
HR-QoL – T0	38.65	8.24
HR-QoL – T1	32.75	21.92
HR-QoL – T2	14.6	18.62

*T0 Pre-surgery: 1 month before the surgery

*T1 Post-surgery: 2 weeks after the surgery

*T2 Follow-up: 6 months post-surgery

**Table 2.** Mean and standard deviation for GHS/QoL at each time point.

Global Health Status/QoL	*M*	SD
Global Health Status/QoL –T0	71.61	20.89
Global Health Status/QoL – T1	62.91	19.98
Global Health Status/QoL – T2	75.66	19.12

*T0 Pre-surgery: 1 month before the surgery

*T1 Post-surgery: 2 weeks after the surgery

*T2 Follow-up: 6 months post-surgery
